# Crystal structure and molecular dynamics of human POLDIP2, a multifaceted adaptor protein in metabolism and genome stability

**DOI:** 10.1002/pro.4085

**Published:** 2021-05-10

**Authors:** Anastasija A. Kulik, Klaudia K. Maruszczak, Dana C. Thomas, Naomi L. A. Nabi‐Aldridge, Martin Carr, Richard J. Bingham, Christopher D. O. Cooper

**Affiliations:** ^1^ Department of Biological and Geographical Sciences School of Applied Sciences, University of Huddersfield Huddersfield UK; ^2^ Present address: Astbury Centre for Structural Molecular Biology School of Molecular and Cellular Biology, University of Leeds Leeds LS2 9JT UK; ^3^ Present address: Interfaculty Institute of Biochemistry University of Tübingen Tübingen 72074 Germany

**Keywords:** DNA polymerase, PCNA, PDIP38, POLDIP2, PrimPol, protein structure

## Abstract

Polymerase δ‐interacting protein 2 (POLDIP2, PDIP38) is a multifaceted, “moonlighting” protein, involved in binding protein partners from many different cellular processes, including mitochondrial metabolism and DNA replication and repair. How POLDIP2 interacts with many different proteins is unknown. Towards this goal, we present the crystal structure of POLDIP2 to 2.8 Å, which exhibited a compact two‐domain β‐strand‐rich globular structure, confirmed by circular dichroism and small angle X‐ray scattering approaches. POLDIP2 comprised canonical DUF525 and YccV domains, but with a conserved domain linker packed tightly, resulting in an “extended” YccV module. A central channel was observed, which we hypothesize could influence structural changes potentially mediated by redox conditions, following observation of a modified cysteine residue in the channel. Unstructured regions were rebuilt by ab initio modelling to generate a model of full‐length POLDIP2. Molecular dynamics simulations revealed a highly dynamic N‐terminal region tethered to the YccV‐domain by an extended linker, potentially facilitating interactions with distal binding partners. Models of POLDIP2 complexed with two of its partners, PrimPol and PCNA, indicated that dynamic flexibility of the POLDIP2 N‐terminus and loop regions likely mediate protein interactions.

## Abstract 2

PDB Code(s): 6Z9C;

## 1 INTRODUCTION

Biochemical processes rarely exist in isolation in the cellular environment. Most processes comprise bridging molecules such as proteins or small organic compounds, that integrate pathways to maintain cellular homeostasis. Bridging proteins need to interact with a wide variety of partners and how they maintain such structural plasticity is unknown. One such example is POLDIP2 (polymerase delta‐interacting protein 2, PDIP38), a poorly characterized protein involved in diverse processes including genome stability, reactive oxygen species (ROS) signaling and mitochondrial metabolism.^1^, ^2^

POLDIP2 was first identified as a partner of the p50 subunit of DNA polymerase δ (Polδ).^3^ It is ubiquitously expressed and occupies various sub‐cellular localizations, depending on cell type.^1^ The N‐terminal domain (NTD) of POLDIP2 comprises of 50 mostly unstructured residues, including a 35 amino acid mitochondrial targeting sequence, with the NTD cleaved off during mitochondrial transit.^4^ This is followed by two predicted globular YccV and ApaG/DUF525 domains, with putative functions in bacterial homologues of hemi‐methylated DNA binding (YccV)^5^ and nucleotide binding (ApaG).^6^ POLDIP2 regulates a number of proteins involved in genome stability,^2^ with POLDIP2 enhancing error‐free bypass of 8‐oxo‐G and other DNA lesions by DNA polymerases Polη and Polλ.^7^, ^8^ POLDIP2 stimulates Polδ by increasing its binding to PCNA, a homotrimeric ring‐shaped processivity factor for DNA synthesis,^7^ potentially by bridging the molecules, with the POLDIP2 NTD required for full stimulation.^2^ PrimPol is an archaeo‐eukaryotic primase involved in genome stability in both the nucleus and mitochondria.^9^ POLDIP2 directly stimulates PrimPol DNA binding, synthesis and processivity during translesion bypass.^8^ Furthermore, POLDIP2 also interacts with multiple mitochondrial components and influences mitochondrial morphology.^10^ POLDIP2 binds to the NADPH oxidase subunit p22^phox^, activating Nox4 production of ROS in vascular smooth muscle cells. This directly regulates cytoskeletal dynamics,^11^ potentially playing a role in neovascularization and response to ischemia and other circulatory disorders. As POLDIP2 stimulates ROS production, its role in genome stability suggests a feedback loop protecting DNA from oxidative damage.^7^ POLDIP2 may also play roles in cancer, associating with proliferation‐related replication proteins^3^ and also its knockdown suppresses lung tumor invasion.^12^

Structural determination of POLDIP2 is required to understand how an otherwise small protein can “moonlight”, by binding and regulating diverse protein partners in processes of significant biomedical interest. Here we report the crystal structure of the POLDIP2^51‐368^ fragment to 2.8 Å, demonstrating that it has a rigid core structure with closely packed extended YccV and DUF525 domains, confirmed by solution scattering and molecular dynamics simulations. We suggest that POLDIP2 interactions with client proteins such as PrimPol and PCNA may require highly dynamic conformational changes of the unstructured N‐terminal region and loops. We also observed a central channel traversing the POLDIP2 core and propose this could act to mediate conformational changes, following the presence of a modified cysteine residue.

## 2 RESULTS

### 2.1 Origin and evolution of POLDIP2

**FIGURE 1:**
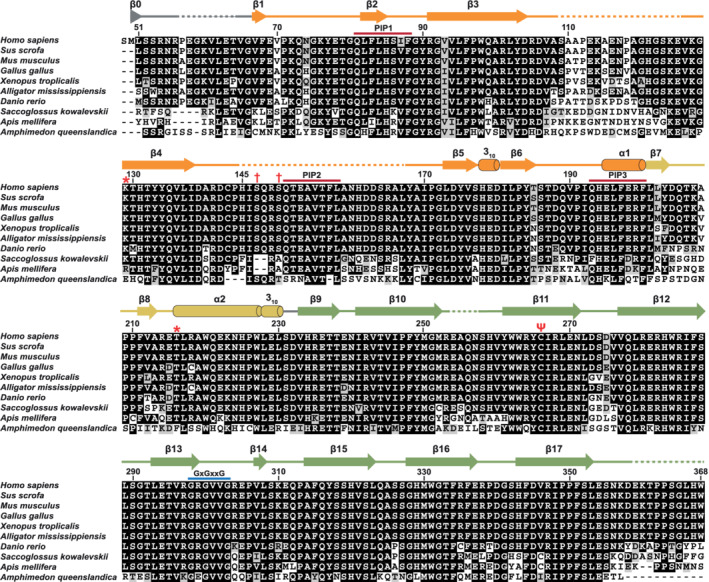
POLDIP2 structural alignment. POLDIP2 protein sequence alignment, with secondary structure assigned from the POLDIP2^51‐368^ structure. Shading reflects amino acid identity/similarity, and secondary structure color reflects domain/region (orange, canonical YccV; yellow, extended YccV; green, DUF525). Residue numbering is above alignment and dashes represent missing electron density; ψ, modified Cys^266^; *, residues crosslinking with PrimPol; †, ATR‐phosphorylated residues; red, PIP motifs; blue, GxGxxG motif

POLDIP2 comprises an unconserved N‐terminal domain, followed by conserved YccV‐like and DUF525 domains (Figures 1 and S1), both thought to derive from bacterial ancestors.^1^ To assist in mapping the evolution of POLDIP2 to its structure, we performed phylogenetic analysis. Reciprocal BLAST searches found no POLDIP2 orthologues in the Fungi/Nucleariida or in eukaryotes outside the Opisthokonta,^1^ but did reveal a single orthologue in the majority of holozoan clades studied, including the Filasterea and Choanoflagellatea (but notably not in the Ichythosporea) (Figures S1 and S2). Phylogenetic analyses showed that although deeper internal branches were poorly supported, the phylogenies were consistent with the vertical inheritance of POLDIP2 since the last common ancestor of Filasterea, Choanoflagellatea, and Metazoa (Figure S2). We hypothesize that POLDIP2 originated from a YccV‐like and DUF525 domain fusion during the opisthokont radiation within the Holozoa. The apparent absence in the ichthyosporean group indicates that POLDIP2 arose within Holozoa after the Ichthyosporea split from the lineage leading to the filastereans, choanoflagellates, and metazoans.

### 2.2 POLDIP2 crystallization and overall structure

We initially screened a number of POLDIP2 expression constructs, with the most suitable being POLDIP2^51‐368^ expressed as an N‐terminal 6xHis/thioredoxin fusion.^13^ This corresponds to the 38 kDa fragment resulting from removal of the mitochondrial targeting sequence (Figure 1).^4^ Hexagonal crystals grew within 24 h which diffracted to 2.8 Å (Figures S3(a,b)). The POLDIP2^51‐368^ structure was solved with molecular replacement, using bacterial HspQ (YccV)^14^ and human FBxo3 (DUF525),^15^ with one molecule in the asymmetric unit and *R*_work_/*R*_free_ factors of 0.216/0.288 respectively, 99.67% completeness and 99.6% of residues in preferred or allowed Ramachandran regions (Table 1, Figure S3(c)). During preparation of this manuscript, another structure of POLDIP2 was independently reported, albeit of a lower resolution (3.4 Å).^16^ This was generally consistent with our structure, although here we also report number of additional observations.

**TABLE 1:** Data collection and structural determination statistics

PDB accession code	6Z9C
Data collection	
Source	D8 venture (Bruker)
Temperature (K)	110
Wavelength (Å)	1.54
Resolution range (Å)	23.88–2.80 (2.95–2.80)
No. of measured reflections	77,725 (6274)
No. of unique reflections	10,246 (1488)
Multiplicity	7.6 (4.2)
Completeness (%)	99.7 (99.7)
Mean *I*/σ(*I*)	14.6 (1.6)
*R*_merge_	0.139 (0.95)
*R*_pim_	0.053 (0.516)
Space group	P62
*a*, *b*, *c* (Å)	120.14, 120.14, 49.52
α, β, γ (°)	90.00, 90.00, 120.00
Refinement	
Resolution range (Å)	23.88–2.80 (2.873–2.800)
No. of reflections	9,711 (702)
Completeness (%)	99.67 (99.87)
*R*_work_	0.216 (0.39)
*R*_free_	0.288 (0.43)
*R*_free_ test set size	524 reflections (5.1%)
Geometry	
RMSD, bond lengths (Å)	0.008
RMSD, bond angles (°)	1.608
Ramachandran plot	
Preferred regions (%)	228 (90.5%)
Allowed regions (%)	23 (9.1%)
Outliers (%)	1 (0.4%)
*B* factors (Å^2^)	
Mean B value	42.6
From Wilson plot	43.7
Total number of atoms	2,184

*Note*: Values in parentheses represent highest resolution shell.

**FIGURE 2:**
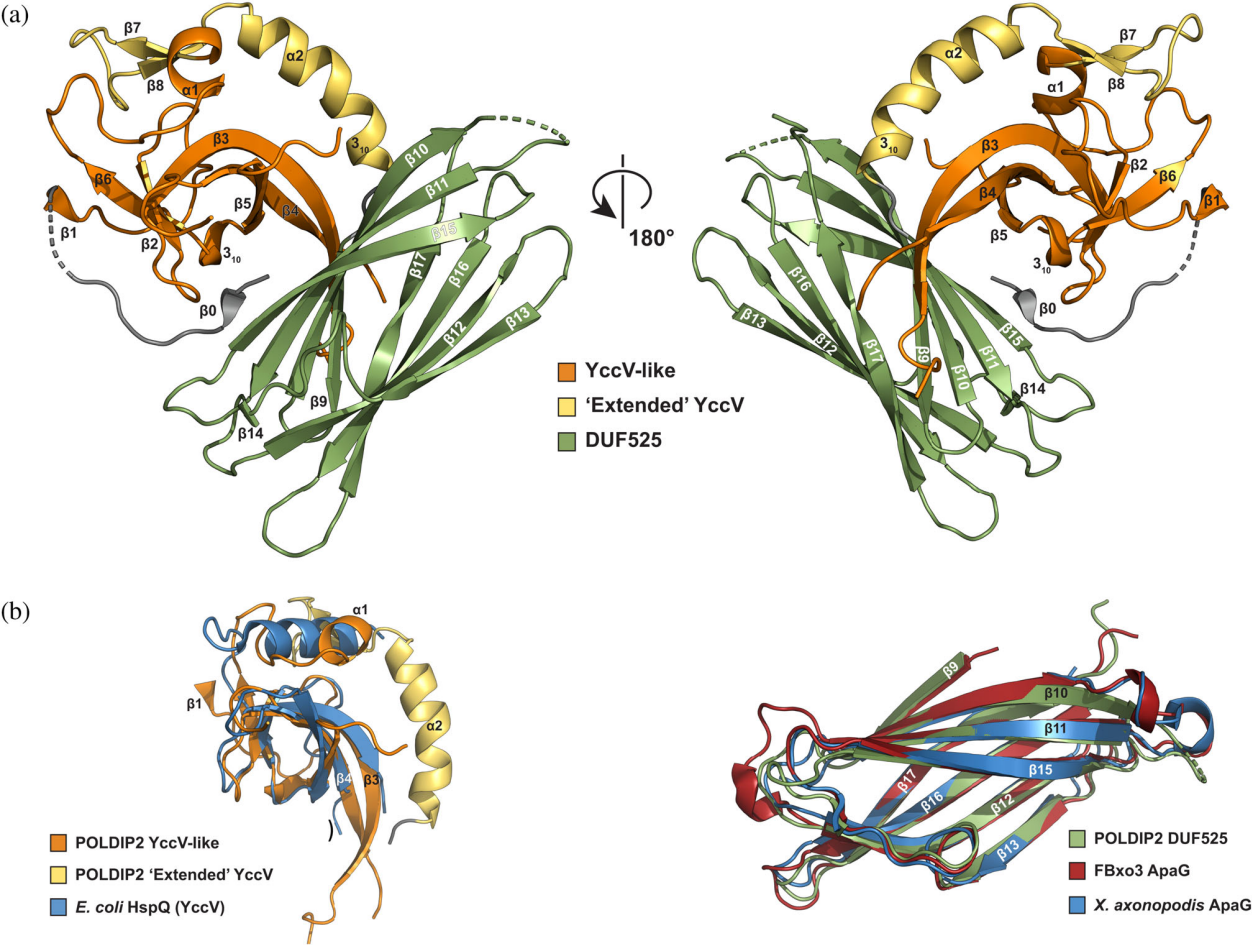
POLDIP2 overall structure. (a) Cartoon representation of the overall POLDIP2^51‐368^ structure (PDB: 6Z9C). Domain colouring: orange, canonical YccV; yellow, extended YccV; green, DUF525). Cylinders, α‐ or 3_10_ helix; arrow, β‐strand; dashed lines, disordered protein regions/missing electron density. (b) POLDIP2 structural superimposition. Left panel: extended YccV domain with *Escherichia coli* HspQ (PDB: 5YCQ; right panel: DUF525 domain with human FBxo3 ApaG (PDB: 5HDW) and *Xanthomonas axonopodis* ApaG (PDB: 2F1E)

The POLDIP2^51‐368^ crystal structure exhibits a globular fold, with the YccV‐like and DUF525 domains juxtaposed on top of each other (Figure 2(a)). The YccV‐like domain (residues 67–200) comprises a five‐membered antiparallel β‐sheet flanked by 3_10_ and C‐terminal α‐helices, reminiscent of a kinked β‐barrel (orange, Figures 1 and 2(a)). This is similar to the canonical YccV fold found in some bacterial proteins such as *Escherichia coli* HspQ (3.4 Å RMSD; Figure 2(b), left panel),^14^ but with extended central β3/β4 strands. A short β1 strand runs antiparallel to β6 at the edge of the β‐sheet, connecting the YccV‐like domain to the N‐terminal region. Two unstructured inter‐strand loops are apparent, with the β4‐β5 loop longer than the equivalent HspQ loop (Figure 2(b), left panel), and the β3‐β4 loop being poorly conserved (Figure 1).

**FIGURE 3:**
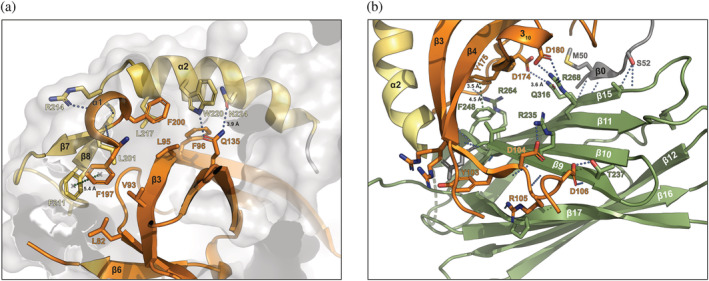
POLDIP2 domain interfaces. (a) Cartoon representation of the YccV/extended region interface. (b) Cartoon representation of the YccV/DUF525 region interface. Key residues are shown in the stick representation. Coloring reflects Figure 2 (orange, YccV; yellow, extended YccV, green, DUF525). Hydrogen bonds are blue dotted lines if <3.4 Å, otherwise inter‐atomic distances are labelled; green dotted lines are π‐π/aromatic interactions

The YccV‐like α1 helix leads immediately into a presumed “linker” region between the YccV and DUF525 domains, comprising the α2/3_10_ helix (residues 201–230). This region is closely packed against the β3 edge of the YccV β‐sheet and the α1 helix, with a significant contact area of ~900 Å^2^ (yellow, Figure 3(a)). Extensive non‐polar contacts are observed here, including a T‐stacking interaction between Phe^197^ and Phe^211^. The YccV α1 helix and β7‐β8 strands exhibit hydrogen bonding, along with a hydrogen bond and weak electrostatic interactions between strand β4 Gln^135^ and the α2 helix (Figure 3(a)). The interactions and sequence conservation of the α2 helix (Figure 1), suggest this region is not an unstructured interdomain linker. Instead, we propose this region is part of an extended YccV (E‐YccV) domain.

The E‐YccV domain is connected by a short linker (Ser^231^‐Asp^232^) to the DUF525 domain (residues 233–368), which exhibits a fibronectin type III/immunoglobulin (β‐sandwich) fold, comprising two opposing four‐stranded antiparallel β‐sheets (Figure 2(a)). This is capped with inter‐strand loops, on one side including a short β‐strand (β14) with no 3_10_ helices, unlike seen for other DUF525 domains (Figure 2(b), right panel). POLDIP2 superimposes well onto related DUF525 proteins (Figure 2(b), right panel), such as FBxo3 (5HDW; 0.7 Å RMSD), the only other protein in the human genome with a DUF525 domain,^15^ and also bacterial ApaG homologues (2F1E; 1.1 Å RMSD). DUF525 has a significant hydrophobic core, with the β‐sandwich opening to form a hydrophobic cleft between strands β13 and β15. DUF525 loop regions were ordered with the exception of the β10‐β11 loop. Part of the N‐terminal loop was structured in the crystal (residues 49–66), comprising the short β0 strand formed partly from Met^50^ (remaining following tag cleavage). β0 hydrogen bonds with the distal β15 strand of the upper β‐sheet (Figure 3(b)), thereby connecting DUF525 to the N‐terminal region (Figure 2(a)). This is in contrast to Strack et al.,^16^ where a few N‐terminal ordered residues ahead of the β1 strand point away from the main body of the structure and β0 is not observed.

### 2.3 The POLDIP2 domain interface reveals a central channel

**FIGURE 4:**
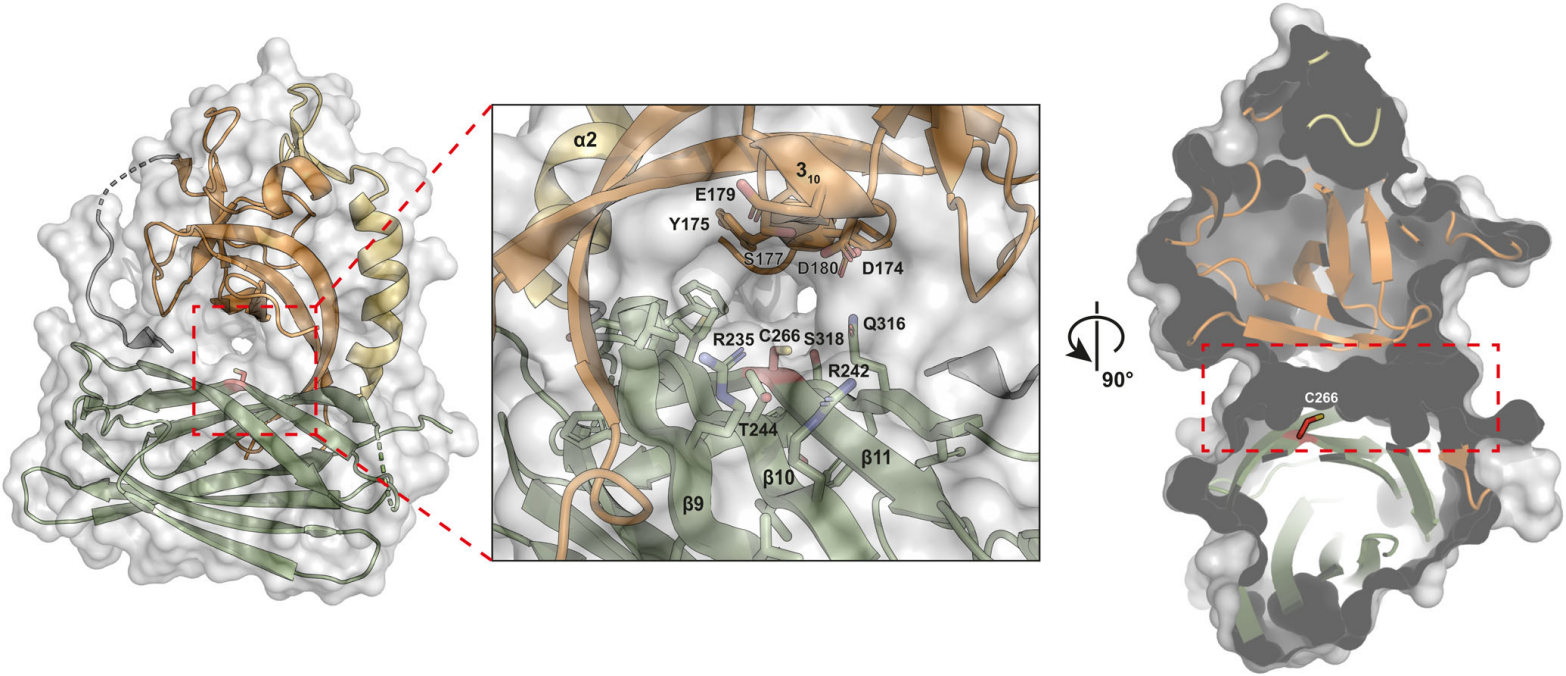
POLDIP2 exhibits a central channel. Left panel: surface and cartoon representation, with channel marked with red dashed box. Inset: magnification of channel showing surface polar/charged residue side chains. Cys^266^ marked in stick form (red). Right panel: cross‐sectional surface representation of channel, marked with red dashed line, displaying Cys^266^ in stick form (red). Cartoon and stick coloring reflects Figure 2 (orange, YccV; yellow, extended YccV, green, DUF525)

The E‐YccV domain is positioned directly on top of the DUF525 β‐sandwich, with the interface comprising predominantly main chain and side chain hydrogen bond contacts, localized to opposite sides of the interface (Figure 3(b)). These are centered around interactions between the β9 strand on the edge of DUF525 and the C‐terminal end of the E‐YccV β3 strand and subsequent loop, and the β11 and β15 strands on the opposing DUF525 face that contacts the N‐terminal β0 strand and areas around the E‐YccV 3_10_ helix and β5 strand. The contact area of the E‐YccV/DUF525 interface is ~1,060 Å^2^, perhaps lower than may be expected, particularly as the intra‐domain E‐YccV interface is ~900 Å^2^. Surprisingly, we did not observe any residue interactions or hydrophobic‐rich internal regions in the central part of the E‐YccV/DUF525 interface. On closer examination we observed an internal cavity traversing the protein at the interface (Figure 4, dashed boxes), lined with predominantly hydrophilic residues contributed from both E‐YccV and DUF525 domains (Figure 4, inset). Clear openings can be seen on opposite sides of the channel, with the narrowest dimensions at ~6.5 Å and ~19 Å for width and breadth, respectively (Figure 4). This channel hence accounts for the lower than expected contact area for E‐YccV/DUF525 domains.

The role of this channel is undetermined, however, we noted the presence of Cys^266^ protruding into the channel (Figure 4, right panel). During structural refinement we observed that Cys^266^ exhibited electron density that could not be accounted for, either by alternative rotamers or by surrounding residues (green, Figure S4, left panel). This additional density was not observed in Strack et al.,^16^ presumably following the lower resolution dataset, although a direct comparison cannot be made as the structure had not been released at the time of writing (PDB**:** 6ZLX). We propose this represents an *in crystallo* cysteine modification, as DNA sequencing confirmed that the electron density at this position did not result from a residue substitution mutation. Protein mass spectrometry also confirmed the expected molecular weight, furthermore, a mass difference reflecting the size of the density was not observed (not shown). This prompted us to speculate that this could reflect an oxidized (or other) post‐crystallization modification of Cys^266^, given its exposure to the cellular environment via the channel. Moreover, POLDIP2 is involved in a number of processes relating to redox status, including ROS generation.^11^ Post‐translational oxidative cysteine modifications are common, including sulfenylation,^17^ often modulating protein structural changes in response to ROS.^18^ Although Cys^266^ does not form hydrogen bonds with surrounding residues, such modifications could change hydrogen bonding patterns, as observed when sulfenic acid was modelled at this position (Figure S4, right panel). This has the potential to influence conformational or dynamic alterations, particularly in the E‐YccV and DUF525 interface (Figure S4). Cys^266^ is conserved in the majority of POLDIP2 orthologues (Figure 1 [ψ], Figure S1), supporting a conserved function. Hence, we speculate that the channel Cys^266^ could be a putative redox sensor, with modifications modulating POLDIP2 interactions or dynamics.

### 2.4 POLDIP2 exhibits a rigid core, with conformational disorder in termini and loops

**FIGURE 5:**
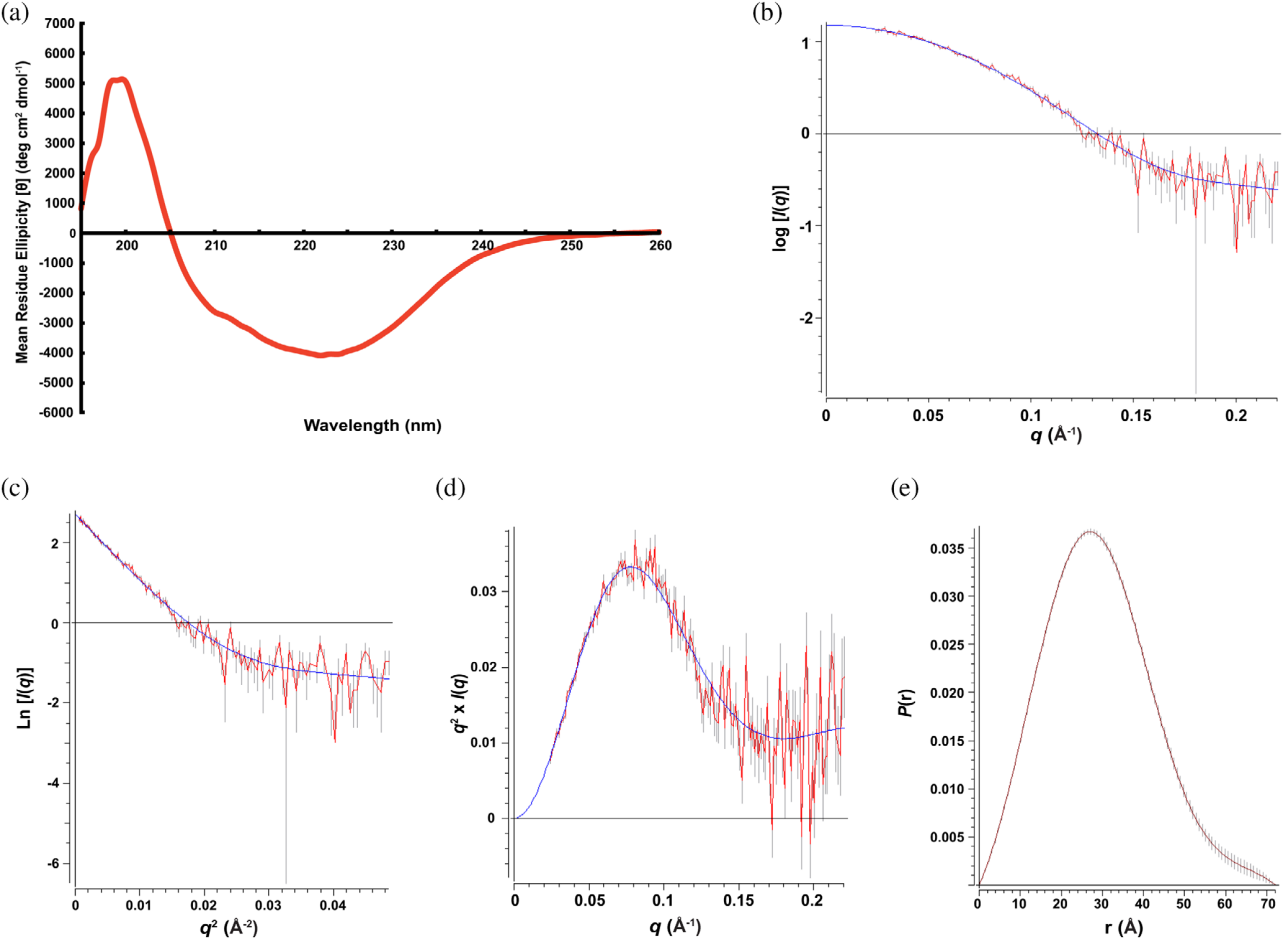
Circular dichroism and SAXS solution structural analysis of POLDIP2^51‐368^. (a) Far‐UV CD analysis normalized to buffer blank. (b) Raw SAXS scattering data following averaging and buffer subtraction in PRIMUS, plotted as log *I*/*q*. (c) Guiner analysis of SAXS data from (b), noting the linear shape at low *q* ^2^ values. (d) Kratky plot (*q* ^2^ × *I*[*q*] vs. *q*) of SAXS data from (b). (e) Distance distribution function *P*(*r*) representation of SAXS data from (b), using a *D*_max_ of 72.22. SAXS analyses: red, raw data; blue, fitted curves; grey, error bars

The high β‐strand content of POLDIP2^51‐368^ was confirmed with circular dichroism analysis (Figure 5(a)). Although we hypothesized that the channel that runs between the E‐YccV and DUF525 domains may have a functional role, it remains a possibility that this channel in the domain interface is a crystal packing artefact. Hence, we performed small angle X‐ray scattering (SAXS) analysis (Figure 5(b)), with POLDIP2^51‐368^ exhibiting a linear Guinier plot at low *q* values, consistent with a monodisperse system (Figure 5(c)). The Kratky plot (Figure 5(d)) is consistent with a globular/partially unfolded structure,^19^ suggesting some contribution from the long, disordered loop regions. The *P*(*r*) distribution indicates a single globular unit (Figure 5(e)), rather than well‐separated subunits displaying multiple maxima,^20^ and the real/reciprocal space *R*_g_ values (22.49/22.72 Å) are consistent with monomeric POLDIP2^51‐368^ dimensions. Hence, the SAXS data are consistent with the crystal asymmetric unit and packing, with E‐YccV associating with DUF525 as a packed unit, supporting the presence of a channel.

**FIGURE 6:**
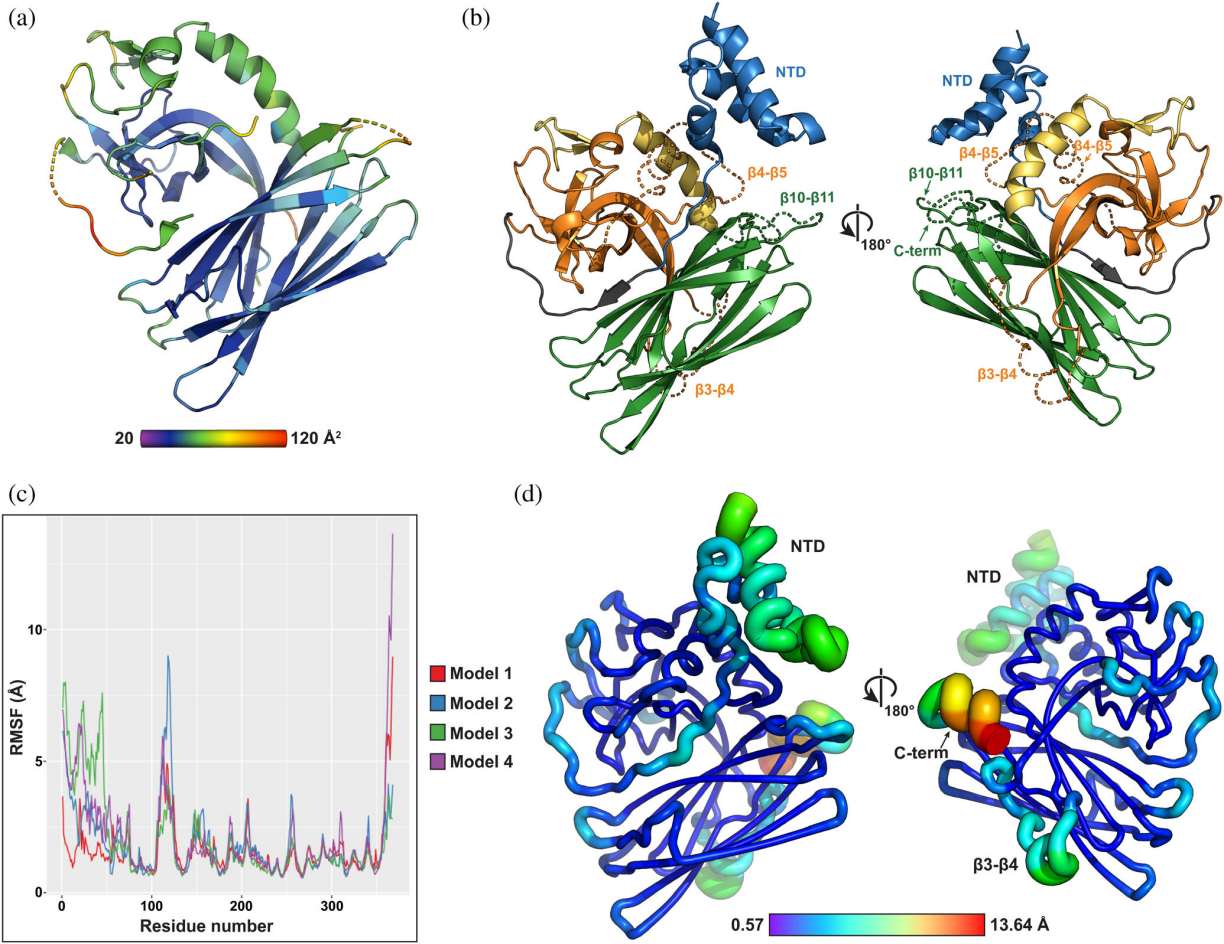
Structural modelling and dynamics of POLDIP2. (a) Temperature factors of POLDIP2^51‐368^ plotted on cartoon representation. (b) Representative Robetta model of POLDIP2^FL^ (#4). Colouring as in Figures 1 and 2(a), excepting N‐terminal domain in blue (NTD) and modelled loops as dashed lines. (c) RMSF analysis of all full‐length Robetta models. Root mean square fluctuation (RMSF, deviation of atoms with respect to reference structure) plotted against residue position. (d) RMSF analysis of 100 ns molecular dynamics simulation of POLDIP2^FL^ model 4, positioned with respect to structures in Figure 2(a). Color scale and chain thickness varies from blue (low fluctuation) to red (high fluctuation)

Crystallographic B factors (Figure 6(a)) indicate POLDIP2^51‐368^ has a rigid core, with significant motion likely for the inter‐strand loop regions and the YccV extension α2/β7‐β8 strands. In addition to the N‐terminal 50 residues excluded from the POLDIP2^51‐368^ expression construct, several external loops are missing from the crystal structure following conformational disorder (Figure 1). These include several interaction sites, such as the NTD contacting PrimPol^8^ and a PCNA‐interacting protein (PIP) box in loop β4‐β5.^3^ To gain further understanding of full‐length POLDIP2 (POLDIP2^FL^), we rebuilt these missing regions in Robetta^21^ using POLDIP2^51‐368^ as a structural template. Five models were generated, each of which differed in the orientation of loops and the NTD (Figure S5(a)). The NTD was modelled as a helical bundle, the arrangement of which varies between the models, presumably reflecting conformational heterogeneity (Figures 6(b) and S5(b)). Model 5 NTD formed a knot topology and was excluded from further analysis. Leu^51^‐Gly^66^ form an extended loop, connecting the NTD to the E‐YccV β1 strand (grey, Figure 6(b)). The crystal structure resolves part of this linker, occupying a narrow groove between the E‐YccV and DUF525 domains. This forms the β0 strand and contacts the DUF525 β‐sheet (Figure 2(a)), potentially stabilizing the interaction between the domains.

To gain insight into possible conformational changes and how these may influence protein–protein interactions, the four POLDIP2^FL^ models were subjected to molecular dynamics simulations. The overall drift from the initial model was evaluated by calculating C_α_ RMSD values over 100 ns simulation, with all four models diverging significantly during the initial stages, and models 1–3 reaching a plateau indicating a stable average conformation, while model 4 continued to diverge (Figure S6(a)). A longer 1000 ns simulation indicated that the N‐terminal α‐helix is very mobile (Figure S6(b)). RMSF analysis revealed that regions of stability and local flexibility were consistent with B‐factors and missing residues (Figures 6(b–d) and S5). All four models revealed high mobilities for loop β3‐β4 and the C‐terminus (Figure 6(c,d)). The NTD has high mobility, tethered to the E‐YccV domain by a linker and stabilized by a short β‐strand against the DUF525 domain. Conformational change could feasibly alter this interface, destabilizing the long linker and freeing the NTD to form more distant interactions.

### 2.5 POLDIP2 surface structural features and interaction motifs

**FIGURE 7:**
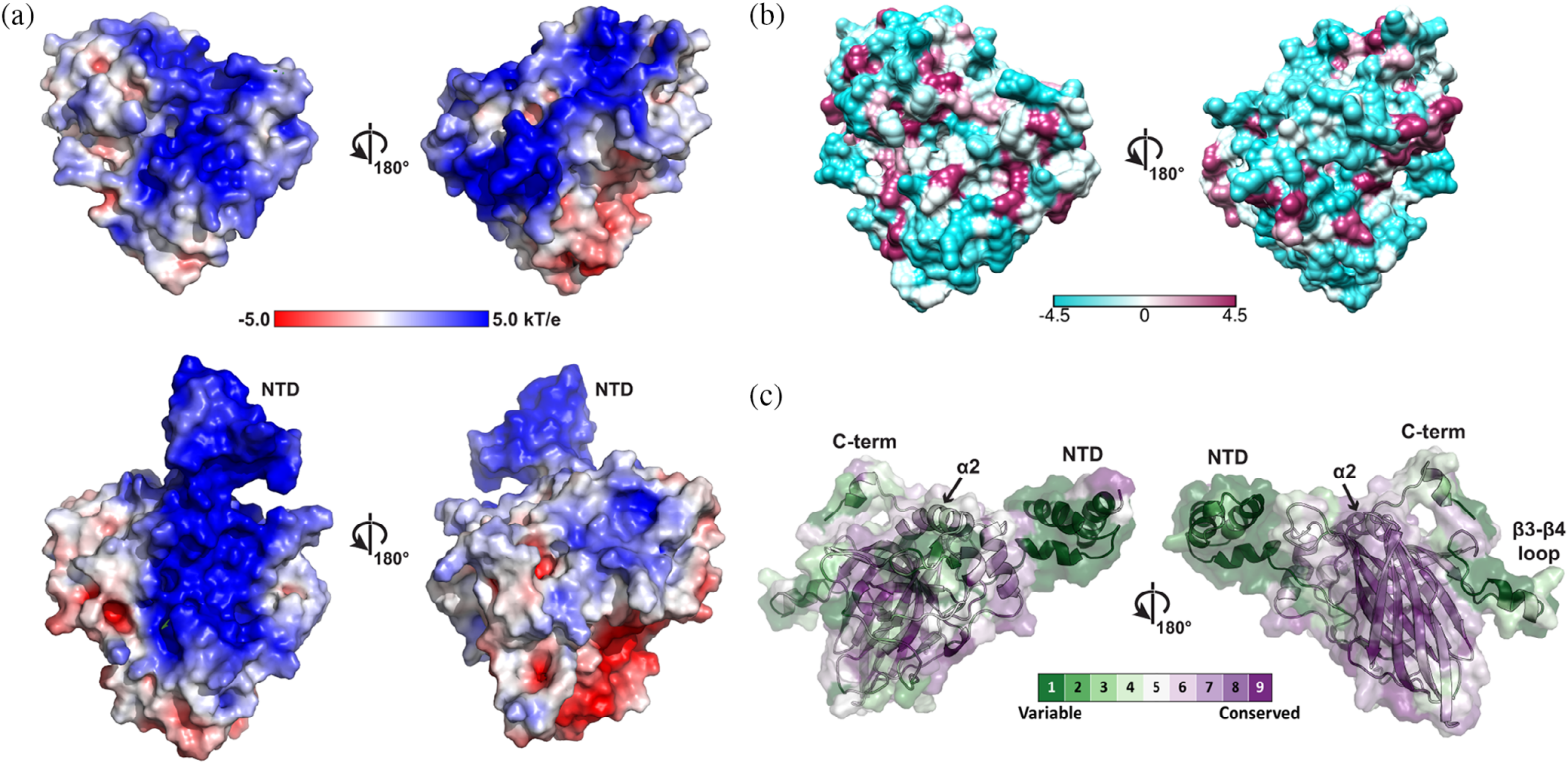
Conserved and surface features of POLDIP2. (a) POLDIP2 electrostatic surface (ABPS^39^), orientated with respect to Figure 2(a). Upper panel: POLDIP2^51‐368^ crystal structure; lower panel full‐length POLDIP2 model 4. NTD, N‐terminal domain. (b) POLDIP2^51‐368^ surface hydrophobicity, orientated with respect to Figure 2(a). Scale bar represents Kyte and Doolittle scale.^53^ (c) Evolutionary conservation calculated with ConSurf mapped onto the POLDIP2^FL^ model 4 (purple, most conserved; green, most variable)

POLDIP2 is observed to interact with a number of proteins from across multiple processes,^1^ but how POLDIP2 exhibits such binding plasticity is unknown. Although the POLDIP2^FL^ model suggests the NTD could be structured, its dynamic nature and heterogeneity of length/sequence composition across the Holozoa (Figure S1) are reminiscent of intrinsically disordered proteins.^22^ This could facilitate binding to many different partners, relying on short linear motifs^23^ such as the N‐terminal helix critical for binding PrimPol^8^ or highly charged regions.^22^ POLDIP2^51‐368^ exhibits basic regions on either face (Figure 7(a)), with the POLDIP2^FL^ model contributing additional positive charge, resulting in a polarized surface as the reverse face containing the β0 strand is less charged overall. This may facilitate generalized electrostatic interactions with partners, perhaps explaining POLDIP2 binding promiscuity.

The POLDIP2^51‐368^ surface also has localized hydrophobic patches on both faces (Figure 7(b)) particularly around the DUF525 cleft as described, potentially contributing to interaction interfaces. The DUF525 β‐sandwich is particularly evolutionarily conserved (Figure 7(c), right), with the β16‐β17 loop directing interaction with the CEACAM1 cell–cell adhesion receptor, binding POLDIP2 to regulate its subcellular localisation.^24^ The most variable regions were the dynamic termini, unstructured β3‐β4 loop and the E‐YccV domain β3‐β4 antiparallel β‐strand, suggesting that flexibility and/or sequence composition rather than structure may be important in directing interactions with some binding partners. DUF525 also contains a conserved glycine‐rich motif (GxGxxG, Figure 1), associated with pyrophosphate or nucleotide binding,^25^ situated beside an arginine‐rich cluster (raspberry, Figure S7(a)).^26^ The GxGxxG motif was not observed to bind nucleotides in DUF525/ApaG homologues,^6^, ^15^ however, both the glycine and arginine‐rich regions are important for PrimPol stimulation.^26^

### 2.6 Structural insights into POLDIP2 interactions with PrimPol

POLDIP2^FL^ stimulates the DNA binding, DNA synthesis and processivity of PrimPol.^8^, ^26^ To gain further insight, we constructed a model of the POLDIP2‐PrimPol complex (Figure S7(a)), where POLDIP2^FL^ and PrimPol^FL^ protomers were manually arranged using known contact points from crosslinking mass spectrometry,^8^ and functional inference from biochemical analyses.^26^ The POLDIP2 NTD N‐terminal helix contacts multiple spatially separated regions around the PrimPol catalytic site^8^ (pink, Figure S7(a)) in keeping with our model, following the highly dynamic nature of the NTD seen both in different POLDIP2 models and simulations (Figures [Link], [Link], and S7(a)). Although one NTD binding site is on the distal side of PrimPol, an interaction is still feasible as the POLDIP2 NTD could potentially unfold and stretch to reach this distal site, particularly if the constraining β0 strand that connects the NTD to the DUF525 domain was destabilized, granting the NTD greater conformational flexibility. Furthermore, the PrimPol disordered loop (brown, Figure S7(a)) is distant from the corresponding crosslinked residues in the E‐YccV domain (Lys^129^ and Thr^216^, Figure 1), to opposite distal faces of the complex. It is feasible that the disordered PrimPol loop could stretch across to interact with the distal face of POLDIP2, as this loop also functionally interacts with the DUF525 region,^25^ which is closer than the E‐YccV crosslinked regions in our model.

Following the requirement of the glycine/arginine‐rich region for PrimPol stimulation to provide additional residues in *trans* to facilitate dNTP binding,^26^ we orientated this region close to where the incoming dNTP binds to the primer/template (p/t) DNA in PrimPol (raspberry, Figure S7(a)). We speculate that this juxtaposition could facilitate PrimPol in binding to DNA, following the strong positive charge in this region of the POLDIP2 DUF525 domain (Figure 7(a)). This may require the POLDIP2 NTD however, as although C‐terminal fragments can stimulate PrimPol DNA synthesis, full stimulation of DNA synthesis and DNA binding by PrimPol is only observed for POLDIP2^FL^,^8^, ^26^ suggesting cooperativity of binding. Although the bacterial homologue to the YccV domain was able to bind hemi‐methylated DNA,^5^ the full POLDIP2 molecule is required for binding to p/t DNA.^26^

### 2.7 POLDIP2 interactions with PCNA may be dependent on conformational flexibility

POLDIP2 was originally identified as a binding partner of the p50 subunit of DNA polymerase δ (Polδ) and also its processivity factor (PCNA).^3^ POLDIP2 stimulates Polδ DNA synthesis and PCNA binding, with the POLDIP2 NTD required for full stimulation.^2^ POLDIP2 contains three putative PCNA‐interacting protein (PIP) box motifs situated in the E‐YccV domain, with PIP1 and PIP3 ordered in the crystal structure, but PIP2 present in the β4‐β5 loop (Figure 1).^3^ None of the PIPs exactly match the canonical motif ([QXXψXXF(F/Y)]; ψ, aliphatic), although it is recognized that “PIP‐like” motifs are still able to bind PCNA.^27^ Mapping the PIPs onto the POLDIP2^51‐368^ structure and POLDIP2^FL^ models juxtaposes PIP1 and PIP3 into close proximity (teal, Figure S7(a)), with PIP2 located nearby in some POLDIP2^FL^ models, but with heterogeneity following β4‐β5 loop flexibility. PIP1 and PIP3 are unlikely to bind PCNA however, as PIP1 is inaccessible being away from the protein surface, and the PIP3 side chains point towards the POLDIP2 core rather than to the PCNA hydrophobic and Q pockets, when superimposed to the DNMT1 PIP box^28^ (Figure S7(a), inset).

PIP2 from the POLDIP2^FL^ models superimposes well to the DNMT1 PIP, and the POLDIP2^FL^ model RMSF values and the lack of electron density in the crystal structure indicate that PIP2 is present on a conformationally flexible loop (Figures 6(c) and S5). Moreover, POLDIP2^FL^ model 1 presents PIP2 as pointing away from the core POLDIP2, which would allow PCNA to access PIP2. We modelled PCNA bound to PIP2 in POLDIP2^FL^ model 1 using the PCNA‐DNMT1 superimposition to juxtapose the protomers (Figure S7(b)). This clearly shows PCNA being able to bind to the POLDIP2 surface by PIP2, and although part of the NTD is trapped within the superimposition, NTD conformational flexibility could allow PCNA to bind unimpeded. Furthermore, when the POLDIP2‐PCNA and POLDIP2‐PrimPol complex models are overlaid (Figure S7(b)), it can be seen that PCNA could occlude PrimPol from interacting with POLDIP2. This may prevent PrimPol accessing both the NTD and glycine/arginine‐rich DUF525 regions, supporting previous observations where although PCNA is not known to interact with PrimPol, PCNA addition to both PrimPol and POLDIP2 caused PrimPol inhibition.^8^

## 3 DISCUSSION

POLDIP2 interacts directly with a large number of proteins involved in wide variety of cellular processes, in particular, DNA polymerases and other replication and repair proteins, such as PrimPol,^8^ Polλ,^7^ Polη^29^ and PCNA.^3^ It is also observed to interact with Polζ (via REV7) and REV1,^29^ Polβ,^30^ and also to partially stimulate Polι,^7^ but it is unknown how POLDIP2 binds to diverse proteins. To this end, we present the crystal structure of POLDIP2, demonstrating that the core protein is a β‐strand rich globular protein, comprising an evolutionarily conserved extended (E‐)YccV domain, juxtaposed on top of a DUF525 β‐sandwich domain. Evolutionary analysis supports fusion of these domains during eukaryotic evolution, occurring soon after the establishment of the Holozoa.

We demonstrate that the POLDIP2 core is stable and rigid, with modelling and simulations suggesting dynamic flexibility of the NTD and loop regions being important in mediating protein interactions. Conformational flexibility of the NTD in particular reconciles previous observations showing it binding to spatially separated regions of PrimPol.^8^ From our models, we speculate that a possible role of the POLDIP2 NTD is to clamp and stabilize a cooperative ternary complex between POLDIP2, p/t DNA and PrimPol, potentially helping to enclose the p/t DNA with its positive charge, to stimulate DNA synthesis by potentiating PrimPol binding to DNA. Our models also support the POLDIP2 PIP2 motif in binding PCNA as it is the only accessible PIP, being on a flexible loop. Modelling also suggests if PCNA binds to PIP2 it could block POLDIP2 from stimulating PrimPol.^8^ Although PrimPol is required for replication fork progression under both normal and DNA‐damaging conditions,^9^ this mutual exclusivity of PCNA/PrimPol binding to PCNA could help prevent aberrant PrimPol recruitment to the replication fork when PCNA is complexed Polδ. POLDIP2 is phosphorylated on Ser^147^ and Ser^150^ by ATR following UV irradiation.^31^ These residues are situated beside PIP2 (Figure 1) and as phosphorylation would add a bulky negatively charged moiety, the binding properties of PIP2 would likely change. Hence, future studies should address if POLDIP2 post‐translational modification can influence its interaction with PCNA.

Although another POLDIP2 structure was reported during preparation of this manuscript,^16^ the data presented here offer additional insights and features not observed in the previous analysis. This includes the presence of a channel traversing the POLDIP2 core which we speculate could house a conformational switch, owing to a putative modification of Cys^266^ contained within the channel and its location at the E‐YccV/DUF525 domain interface. Such a switch could respond to changes in cellular redox potential, as observed for other proteins such as Src kinase.^18^ This is of particular relevance for POLDIP2 as it plays significant roles in redox regulation, including upregulation of ROS production by Nox4,^11^ but also facilitating translesion bypass across oxidized DNA lesions resulting from ROS damage.^7^ Although we anticipate any potential conformational changes to be slight following examination of structure B factors and modelling simulations (Figure 6), these could however still have dramatic effects on the disordered loop and terminal regions on POLDIP2. Small conformational changes could potentially destabilize the weakly hydrogen bonded β0‐β15 antiparallel β‐sheet interactions which connect the β0 N‐terminal region to the C‐terminal DUF525 domain (Figure 2(b), a feature also not present in the POLDIP2 structure from Strack et al.^16^). Destabilizing this region would allow even greater dynamic flexibility of the upstream NTD. Such conformational flexibility of the NTD is likely to be key to many POLDIP2 protein interactions (Figure S7(a)), as discussed for PrimPol. It is possible of course that gross structural rearrangements could occur on POLDIP2 binding to a partner, therefore structural studies on POLDIP2 complexes will be important to test these hypotheses, towards determining how POLDIP2 can act as a central nexus, connecting redox metabolism and genome stability.

## 4 MATERIALS AND METHODS

### 4.1 Cloning and recombinant protein expression and purification

Human POLDIP2^51‐368^ was amplified by PCR from plasmid pETM33,^7^ using Phusion DNA polymerase (New England Biolabs, Ipswich) and primers POLDIP2‐f001 (5'‐TACTTCCAATCCATGCTCTCGTCCCGAAACCGAC‐3′) and POLDIP2‐r000 (5'‐TATCCACCTTTACTGTCACCAGTGAAGGCCTGAGGG‐3′). PCR products were cloned by a ligation independent approach into the pNH‐TrxT expression vector.^13^ Constructs were transformed into *Escherichia coli* BL21 (DE3) Rosetta2™ and cultured at 37°C in Terrific Broth containing 50 μg/ml kanamycin. Expression was induced with 0.1 mM isopropyl‐β‐D‐1‐thiogalactopyranoside and cells incubated overnight at 18°C, prior to harvesting. Cells were resuspended in buffer A (50 mM HEPES pH 7.5, 5% (vol/vol) glycerol, 500 mM NaCl, 10 mM imidazole, 4 mM β‐mercaptoethanol (β‐ME), 0.5 mg/ml lysozyme, 5 U/ml Basemuncher nuclease (Abcam, Cambridge, UK), 1 mM phenylmethylsulphonyl fluoride and 1 mM benzamidine‐HCl) and disrupted by sonication on ice. Lysates were clarified by centrifugation and applied to a Ni‐NTA affinity column (QIAGEN, Hilden, Germany). Columns were washed in buffer A, followed by wash buffer (buffer A with 30 mM imidazole), and eluted in buffer A containing 300 mM imidazole. Fusion tags were cleaved with 6xHis‐tagged TEV protease, with concurrent dialysis in buffer B (20 mM HEPES pH 7.5, 5% (vol/vol) glycerol, 500 mM NaCl, 10 mM imidazole, 4 mM β‐ME), with TEV protease removed by repeated Ni‐NTA affinity column. Pooled fractions were separated by size exclusion using a HiLoad 16/600 S200 column (GE Healthcare, Chicago) in buffer B, with β‐ME substituted for 1 mM DTT. Protein concentration was calculated from OD_280_ using molecular mass and extinction coefficients, and LC/ESI‐TOF mass spectrometry used to confirm protein identity. Proteins were concentrated using 10 kDa MWCO centrifugal concentrators (VIVAproducts, Littleton).

### 4.2 Crystallization and structural determination

POLDIP2^51‐368^ protein (20 mg/ml) was crystallized at 293 K, using a ratio of 1:1 with mother liquor in 2 μl sitting drops. Crystals were obtained in 0.2 M calcium acetate, 0.1 M sodium cacodylate, 40% PEG300, pH 6.5 and flash‐cooled directly in liquid nitrogen without addition of further cryoprotectant. X‐ray diffraction data were collected using a Bruker D8 Venture source coupled with a CMOS‐PHOTON II detector (Bruker). Data reduction was performed using Proteum3 (Bruker) and SCALA.^32^ Structure solution was by molecular replacement in Phaser^33^ using models based on 5HDW (DUF525 domain) and 5YCQ (YccV domain). Search models were modified by deletion of surface loops and removal of side chains by PDBSET.^34^ Density modification was conducted using DM before automatic chain tracing in BUCCANEER.^35^ Refinement alternated between real‐space refinement in *Coot* ^36^ and reciprocal space refinement in REFMAC5. Data collection and refinement statistics are given in Table 1, with Ramachandran analysis performed in RAMPAGE.^37^ Structural interfaces were determined in PISA^38^ and electrostatic calculations were performed with APBS.^39^

### 4.3 Molecular dynamics simulations and protein modelling

The N‐terminus (residues 1–50) and several external loops missing from the POLDIP2^51‐368^ crystal structure were rebuilt using the structure as a template by comparative modelling in Robetta,^21^ generating five models. The full‐length PrimPol model was generated in Robetta using the PrimPol^1‐354^ crystal structure (5L2X).^40^ POLDIP2 models were used to initiate five separate 100 ns simulations using Gromacs^41^ with CHARMM27 all atom force field (CHARMM22 plus CMAP for proteins). Models were solvated in a dodecahedron box with a minimum distance to edge of 1.0 nm using the TIP3P water model with water coordinates from the SPC system. NaCl ions were added to 0.1 M and the system was neutralized by addition of additional ions. Structures were minimized by steepest descent until the maximum force was <1000 kJ/mol/nm. The system was then heated to 300 K using NVT conditions followed by stabilization of pressure under NPT conditions. Long‐range electrostatic interactions were calculated using Particle Mesh Ewald method with 1 nm cutoff. Production runs were either 100 or 1000 ns with Berendsen temperature coupling and Parrinello–Rahman pressure coupling. Trajectories were analysed using the Bio3D package in R.^42^

### 4.4 Small angle X‐ray scattering

POLDIP2^51‐368^ protein was dialyzed into 20 mM HEPES (pH 7.5), 5% (vol/vol) glycerol, 500 mM NaCl, overnight at 4°C. Scattering data was collected using a Nanostar Vantec 2000 instrument (Bruker) from 100 μl of 3.4 mg/ml POLDIP2^51‐368^ in a 1.5 mm bore quartz glass capillary, under vacuum for 10 exposures of 1800 s. Data were averaged and buffer scattering was subtracted using PRIMUS in the ATSAS package^43^ and deposited in SASBDB^44^ under accession SASDK76. *R*_g_ values were calculated using the Guiner approximation in PRIMUS.

### 4.5 Circular dichroism spectroscopy

Circular dichroism experiments were performed on a Chirascan spectrophotometer (Applied Photophysics, Beverley). Far‐UV spectra (180–260 nm) were obtained in a 1 mm pathlength quartz cuvette, with 0.2 ml of 0.45 mg/ml protein (in 50 mM sodium phosphate, pH 7.5) at 5°C. Four spectra were averaged with 1 nm resolution, 1 s response time, 2 nm bandwidth, with buffer blank spectra subtracted. Data were analysed with DICHROWEB.^45^

### 4.6 Sequence and phylogenetic analysis

POLDIP2 orthologues were found using a reciprocal homology approach using BLASTP,^46^ with *Homo sapiens* POLDIP2 (NP_056399.1) used as a query against genomes in the NCBI GenBank database, or the BLAST utility at the Mnemiopsis Genome Project Portal.^47^ Protein sequence alignment was in MAFFT v7.450^48^ and ambiguously aligned regions and gaps removed with Gblocks.^49^ A Bayesian inference phylogeny was created in MrBayes 3.2.6^50^ and run with a mixed amino acid model and a four‐category gamma distribution. The MCMC analyses consisted of 5,000,000 generations using two parallel chain sets at default temperatures. Sampling frequency was 1000, with a burn in value of 1250. A maximum likelihood (ML) phylogeny was created with raxmlGUI 2.0,^51^ using 1000 bootstrap replicates. The ML tree was generated from 100 starting parsimony trees, using the PROTCAT model and the JTT amino acid substitution matrix. Empirical amino acid frequencies were used in the ML analysis. Residue evolutionary conservation was calculated with ConSurf.^52^

## AUTHOR CONTRIBUTIONS

**Anastasija A. Kulik:** Investigation. **Klaudia K. Maruszczak:** Investigation. **Dana C. Thomas:** Investigation. **Naomi L. A. Nabi‐Aldridge:** Investigation. **Martin Carr:** Investigation; methodology; visualization. **Richard. Bingham:** Investigation; methodology; visualization. **Christopher D. O. Cooper:** Conceptualization; investigation; methodology; supervision; visualization.

## CONFLICT OF INTEREST

The authors declare no conflicts of interest.

## Supporting information


**Figure S1**: Protein sequence alignment of POLDIP2 orthologues from major eukaryotic groups, elucidated from reciprocal BLASTP searches. Alignment was generated using MAFFT in Geneious PRIME and visualised in Jalview. Residue position is above the alignment, residue colouring is according to ClustalX, and panel below the alignment is Jalview conservation score. Sequence and GenBank identifiers were: *Homo sapiens* (NP_056399.1), *Pan troglodytes* (XP_016787681.1), *Macaca ulatta* (NP_001248642.1), *Callithrix jacchus* (XP_008995455.2), *Canis lupus familiaris* (NP_001240832.1), *Sus scrofa* (XP_003358222.2), *Pteropus vampyrus* (XP_011358283.1), *Tursiops truncatus* (XP_019782617.1), *Loxodonta africana* (XP_003416901.1), *Equus caballus* (XP_023508851.1), *Mus musculus* (NP_080665.1), *Monodelphis domestica* (XP_001368593.1), *Ornithorhynchus anatinus* (XP_028938338.1), *Gallus gallus* (NP_001304285.1), *Taeniopygia guttata* (NP_001232073.1), *Paroedura picta* (GCF45383.1), *Alligator mississippiensis* (XP_019345287.1), *Nanorana parkeri* (XP_018412666.1), *Xenopus tropicalis* (NP_001017098.1), *Danio rerio* (NP_997879.1), *Takifugu rubripes* (XP_003968776.1), *Latimeria halumnae* (XP_006011053.1), *Callorhinchus milii* (XP_007894669.1), *Petromyzon marinus* (XP_032823499.1), *Ciona intestinalis* (XP_002121208.2), *Branchiostoma belcheri* (XP_019641134.1), *Anneissia japonica* (XP_033123282.1), *Saccoglossus kowalevskii* (XP_006824194.1), *Priapulus caudatus* (XP_014665443.1), *Crassostrea gigas* (XP_011415219.2), *Helobdella robusta* (XP_009026644.1), *Lingula anatina* (XP_013400176.1), *Brachionus plicatilis* (RNA06801.1), *Opisthorchis felineus* (TGZ54899.1), *Drosophila melanogaster* (NP_649540.1), *Apis mellifera* (XP_006559902.2), *Caenorhabditis elegans* (NP_498703.2), *Hypsibius dujardini* (OQV20687.1), *Nematostella vectensis* (XP_032226378.1), *Trichoplax adhaerens* (XP_002114156.1), *Amphimedon queenslandica* (XP_019848720.1), *Mnemiopsis leidyi* (AGCP01016403), *Salpingoeca rosetta* (XP_004988627.1), *Capsaspora owczarzaki* (XP_004349208.2).

**Figure S2**: Bayesian inference phylogeny of POLDIP2 protein sequences. The phylogeny was constructed from 368 aligned amino acid positions using the PROTCAT model, with the JTT substitution matrix, and estimated amino acid frequencies. Values for biPP and mlBP are shown above and below the branches respectively. 1.00 biPP and 100% mlBP are both denoted by “*”. Values <70% mlBP and < 0.97 biPP are denoted by “‐”. The scale bar represents the number of substitutions per site. Colour code: Brown: Filasterea; Light Blue: Choanoflagellatea; Purple: Porifera; Pink: Ctenophora; Orange: Placozoa; Dark Blue: Cnidaria; Green: Protostomia; Red: Deuterostomia.

**Figure S3**: Crystallisation and data collection of POLDIP2^51‐368^. (A) protein crystals of POLDIP2^51‐368^, appearing after 1 week. (B) X‐ray diffraction pattern of POLDIP2^51‐368^ crystal. (C) Ramachandran plot of the ψ/ϕ main chain angles from the solved structure of POLDIP2^51‐368^ (PDB: 6Z9C), with 99.6% of residues in preferred or allowed regions.

**Figure S4**: Modification of Cys^266^ in the POLDIP2^51‐368^ structure. Left panel: electron density map of POLDIP2 in the vicinity of the channel Cys^266^, with *F*_*o*_‐*F*_*c*_ (green) contoured at 3.0 RMSD and 2*F*_*o*_ *‐F*_*c*_ (blue) contoured at 1.6 RMSD. Right panel: modelling of Cys^266^ (stick, yellow) and two potential conformations of Cys^266^ modified with sulfenic acid (stick, yellow/red), and resulting potential inter‐residue distances. Key residues are shown in the stick representation. Hydrogen bonds are represented as blue dotted lines if <3.4 Å, otherwise inter‐atomic distances are labelled; red spheres are structured water molecules.

**Figure S5**: Structural representation and molecular dynamics simulations for all full‐length POLDIP2 models. (A) cartoon representation of Robetta models. Domain colouring as in Figures 1 and 2(a), excepting N‐terminal domain in blue cartoon representation (NTD) and modelled loops as dashed lines. C‐term, C‐terminus. (B) 100 ns molecular dynamics simulation of Robetta models (orientated with respect to respective model in panel (A), with colour scale and chain thickness representing RMSD.

**Figure S6**: POLDIP2^FL^ molecular dynamics time simulations. (A) root mean square deviation (RMSD, deviation of model over time from reference position) simulation over 100 ns. (B) cartoon representation of NTD dynamics of POLDIP2^FL^ model 41,000 ns simulation. Core structure is model 4 at time 0 ns, with NTD structures superimposed from five time points (N, N‐terminus).

**Figure S7**: Structural modelling of the POLDIP2^FL^‐PrimPol^FL^ protein complex. (A) Structural model of POLDIP2^FL^ interactions with PrimPol^FL^. Molecules are separated to illustrate interactions, with matching colours and connecting lines representing functional (raspberry, dotted lines) or crosslinking mass spectrometric (pink/brown, dashed lines) studies. Protein structures were derived using Robetta from the POLDIP2^51‐368^ crystal structure determined herein (6Z9C), and the PrimPol^1‐354^ crystal structure (5L2X). POLDIP2^FL^ model 4 (Figure 6) was used as a representative model, with 1,000 ns simulation (Figure S5b) timepoints superimposed for the NTD to illustrate temporal heterogeneity. Dark green/teal, potential PIP boxes in POLDIP2^FL^ model 4. Inset: PIP boxes from the POLDIP2^51‐368^ crystal structure and POLDIP2^FL^ models 1 and 4, superimposed onto the DNMT1 PIP box bound to human PCNA (63KA), with consensus residues represented as sticks. (B) Structural model of human PCNA (63KA) abrogating POLDIP2^FL^‐PrimPol^FL^ interactions by steric occlusion, with POLDIP2 and PrimPol separated for illustrative purposes. The human PCNA‐DNMT1 PIP‐peptide complex (teal green) was superimposed against the POLDIP2^FL^ model 1 PIP2 box, with POLDIP2^FL^‐domain colouring as in Figures 1 and 2(a). PrimPol is positioned according to (A) but distanced, to demonstrate mutual exclusivity of PCNA/PrimPol positioning when juxtaposed to POLDIP2.
