# Supplementary figures and images for: Crystal structure and molecular dynamics of human POLDIP2, a multifaceted adaptor protein in metabolism and genome stability
Source: Protein Sci. 2021 May 10;30(6):1196–209. doi: 10.1002/pro.4085 (PMC8138528; doi:10.1002/pro.4085)

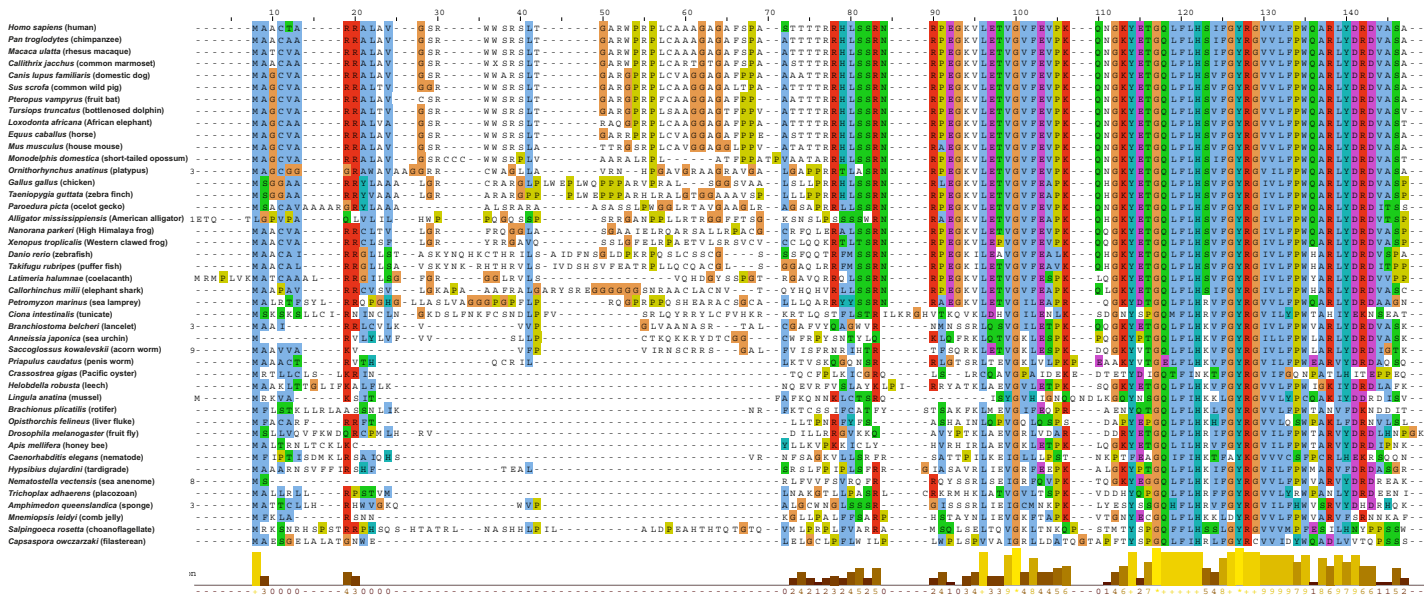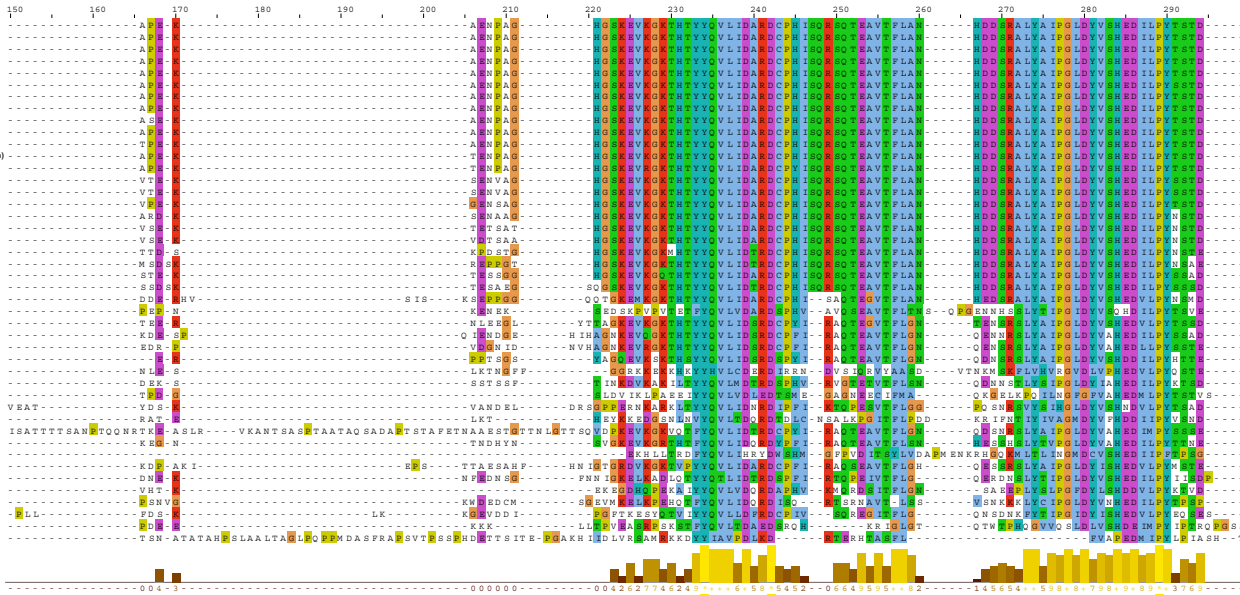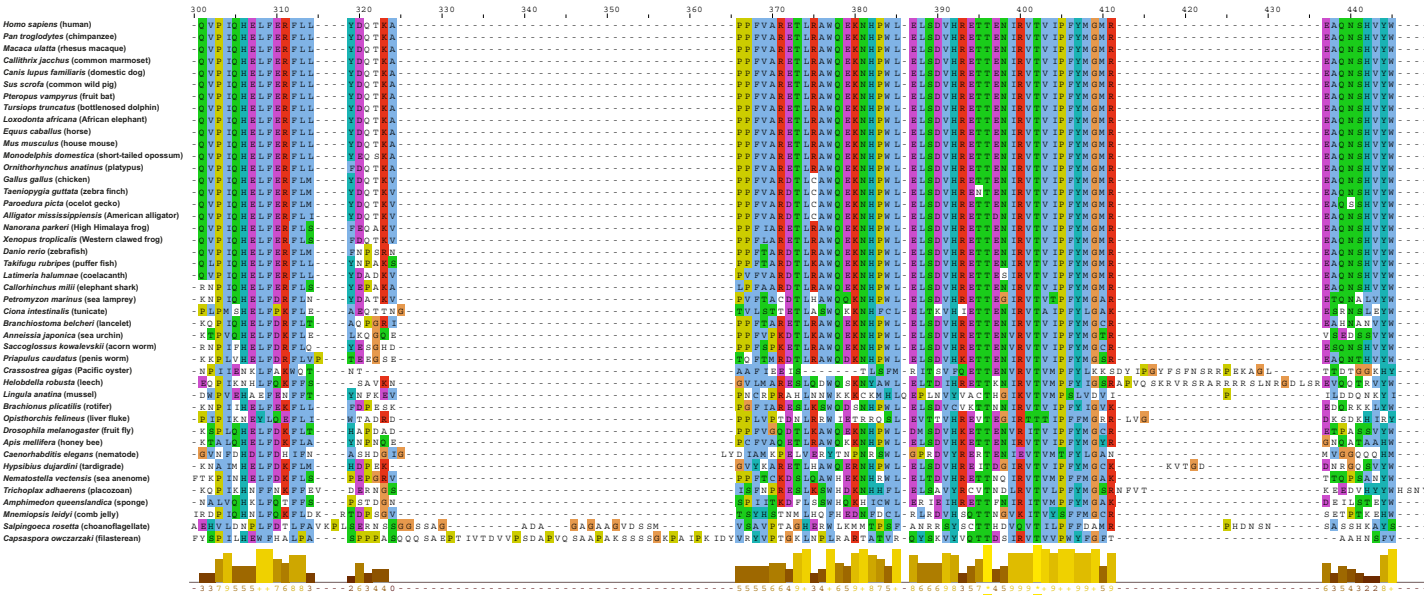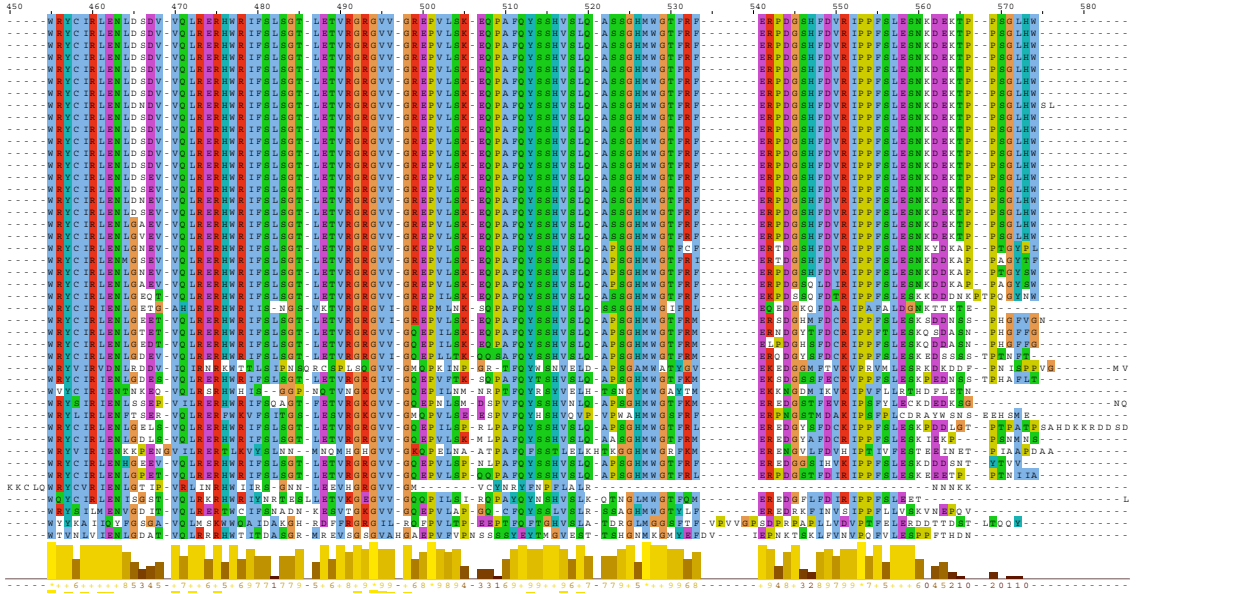

Supplement: Supplementary file 1 — Figure S1: Protein sequence alignment of POLDIP2 orthologues from major eukaryotic groups, elucidated from reciprocal BLASTP searches. Alignment was generated using MAFFT in Geneious PRIME and visualised in Jalview. Residue position is above the alignment, residue colouring is according to ClustalX, and panel below the alignment is Jalview conservation score. Sequence and GenBank identifiers were: Homo sapiens (NP_056399.1), Pan troglodytes (XP_016787681.1), Macaca ulatta (NP_001248642.1), Callithrix jacchus (XP_008995455.2), Canis lupus familiaris (NP_001240832.1), Sus scrofa (XP_003358222.2), Pteropus vampyrus (XP_011358283.1), Tursiops truncatus (XP_019782617.1), Loxodonta africana (XP_003416901.1), Equus caballus (XP_023508851.1), Mus musculus (NP_080665.1), Monodelphis domestica (XP_001368593.1), Ornithorhynchus anatinus (XP_028938338.1), Gallus gallus (NP_001304285.1), Taeniopygia guttata (NP_001232073.1), Paroedura picta (GCF45383.1), Alligator mississippiensis (XP_019345287.1), Nanorana parkeri (XP_018412666.1), Xenopus tropicalis (NP_001017098.1), Danio rerio (NP_997879.1), Takifugu rubripes (XP_003968776.1), Latimeria halumnae (XP_006011053.1), Callorhinchus milii (XP_007894669.1), Petromyzon marinus (XP_032823499.1), Ciona intestinalis (XP_002121208.2), Branchiostoma belcheri (XP_019641134.1), Anneissia japonica (XP_033123282.1), Saccoglossus kowalevskii (XP_006824194.1), Priapulus caudatus (XP_014665443.1), Crassostrea gigas (XP_011415219.2), Helobdella robusta (XP_009026644.1), Lingula anatina (XP_013400176.1), Brachionus plicatilis (RNA06801.1), Opisthorchis felineus (TGZ54899.1), Drosophila melanogaster (NP_649540.1), Apis mellifera (XP_006559902.2), Caenorhabditis elegans (NP_498703.2), Hypsibius dujardini (OQV20687.1), Nematostella vectensis (XP_032226378.1), Trichoplax adhaerens (XP_002114156.1), Amphimedon queenslandica (XP_019848720.1), Mnemiopsis leidyi (AGCP01016403), Salpingoeca rosetta (XP_004988627.1), Capsaspora owczarzaki (XP_004349208.2). [file PRO-30-1196-s006.pdf]

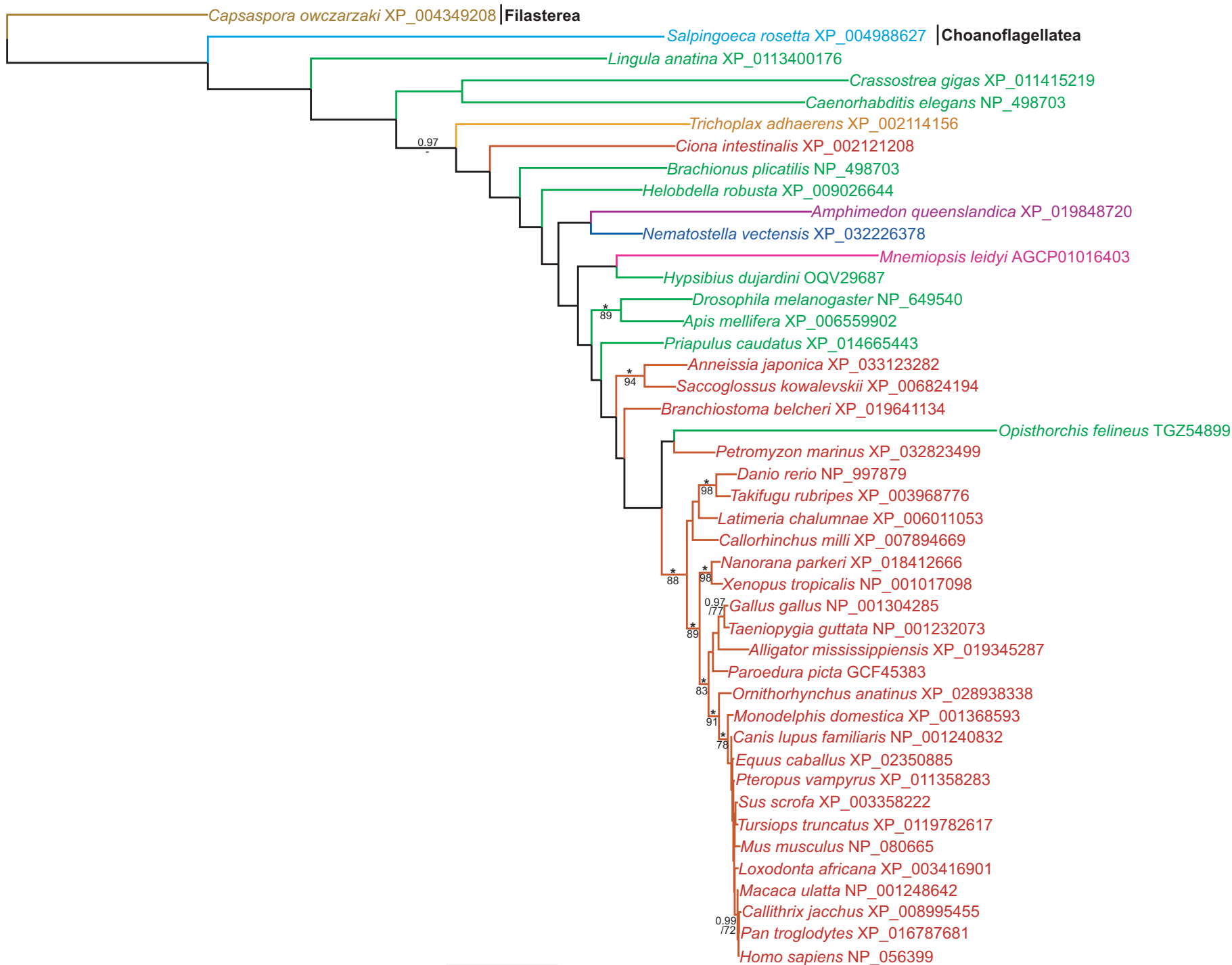

Metazoa

0.6

Supplement: Supplementary file 2 — Figure S2: Bayesian inference phylogeny of POLDIP2 protein sequences. The phylogeny was constructed from 368 aligned amino acid positions using the PROTCAT model, with the JTT substitution matrix, and estimated amino acid frequencies. Values for biPP and mlBP are shown above and below the branches respectively. 1.00 biPP and 100% mlBP are both denoted by “*”. Values <70% mlBP and < 0.97 biPP are denoted by “‐”. The scale bar represents the number of substitutions per site. Colour code: Brown: Filasterea; Light Blue: Choanoflagellatea; Purple: Porifera; Pink: Ctenophora; Orange: Placozoa; Dark Blue: Cnidaria; Green: Protostomia; Red: Deuterostomia. [file PRO-30-1196-s007.pdf]

A

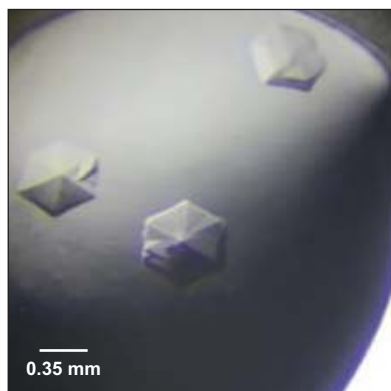

B

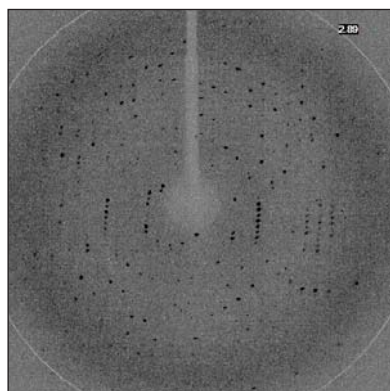

C

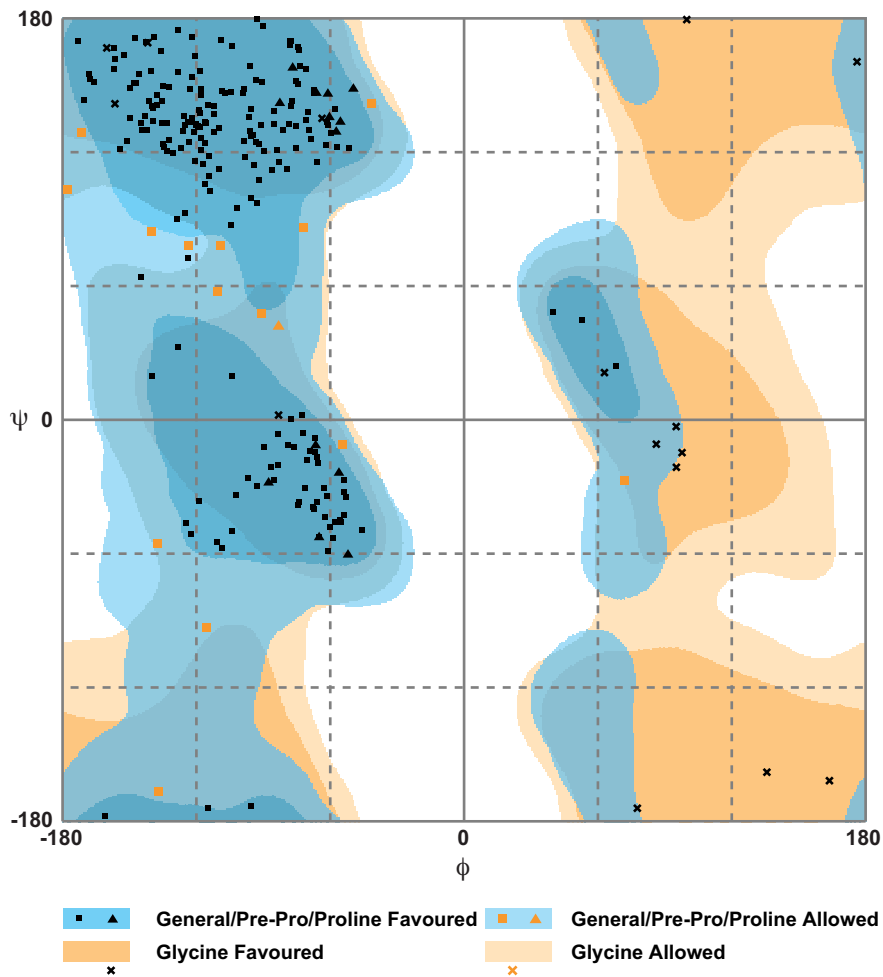

Supplement: Supplementary file 3 — Figure S3: Crystallisation and data collection of POLDIP251‐368. (A) protein crystals of POLDIP251‐368, appearing after 1 week. (B) X‐ray diffraction pattern of POLDIP251‐368 crystal. (C) Ramachandran plot of the ψ/ϕ main chain angles from the solved structure of POLDIP251‐368 (PDB: 6Z9C), with 99.6% of residues in preferred or allowed regions. [file PRO-30-1196-s003.pdf]

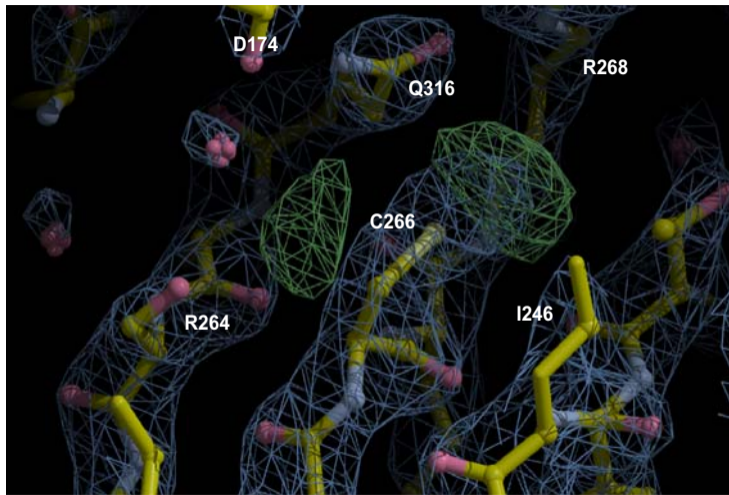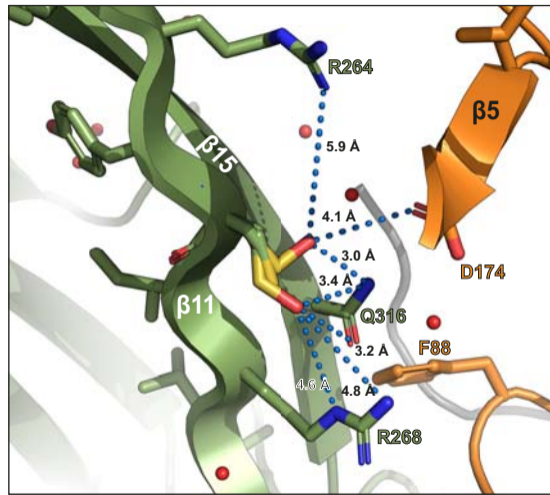

Supplement: Supplementary file 4 — Figure S4: Modification of Cys266 in the POLDIP251‐368 structure. Left panel: electron density map of POLDIP2 in the vicinity of the channel Cys266, with F o‐F c (green) contoured at 3.0 RMSD and 2F o ‐F c (blue) contoured at 1.6 RMSD. Right panel: modelling of Cys266 (stick, yellow) and two potential conformations of Cys266 modified with sulfenic acid (stick, yellow/red), and resulting potential inter‐residue distances. Key residues are shown in the stick representation. Hydrogen bonds are represented as blue dotted lines if <3.4 Å, otherwise inter‐atomic distances are labelled; red spheres are structured water molecules. [file PRO-30-1196-s005.pdf]

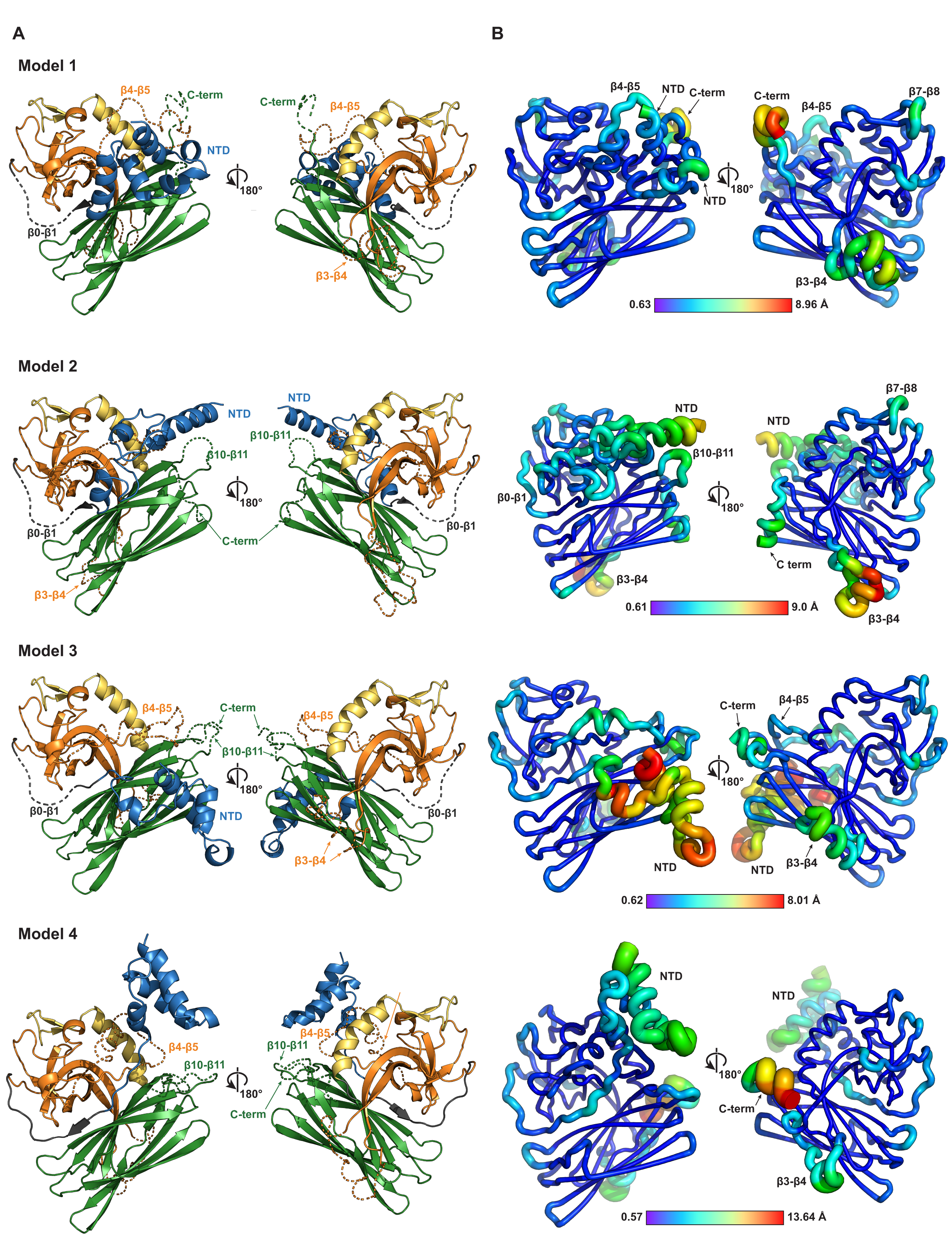

Supplement: Supplementary file 5 — Figure S5: Structural representation and molecular dynamics simulations for all full‐length POLDIP2 models. (A) cartoon representation of Robetta models. Domain colouring as in Figures 1 and 2(a), excepting N‐terminal domain in blue cartoon representation (NTD) and modelled loops as dashed lines. C‐term, C‐terminus. (B) 100 ns molecular dynamics simulation of Robetta models (orientated with respect to respective model in panel (A), with colour scale and chain thickness representing RMSD. [file PRO-30-1196-s004.tif]

**A**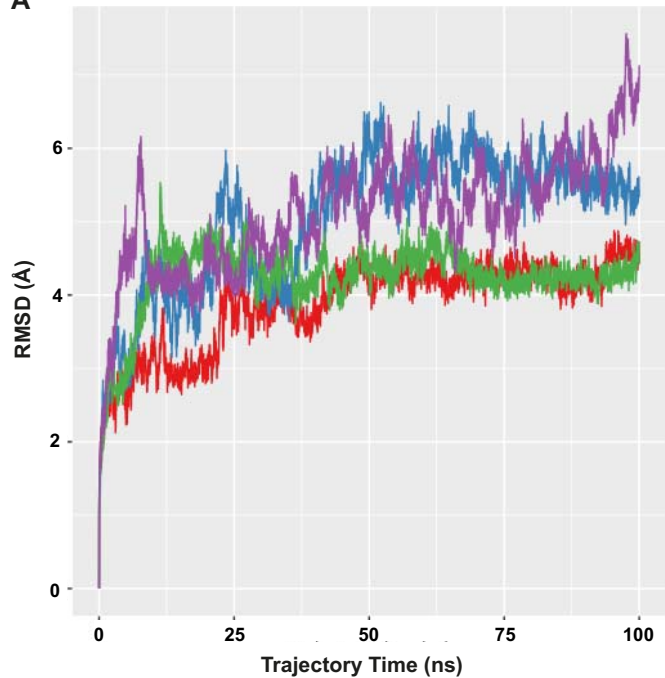**B**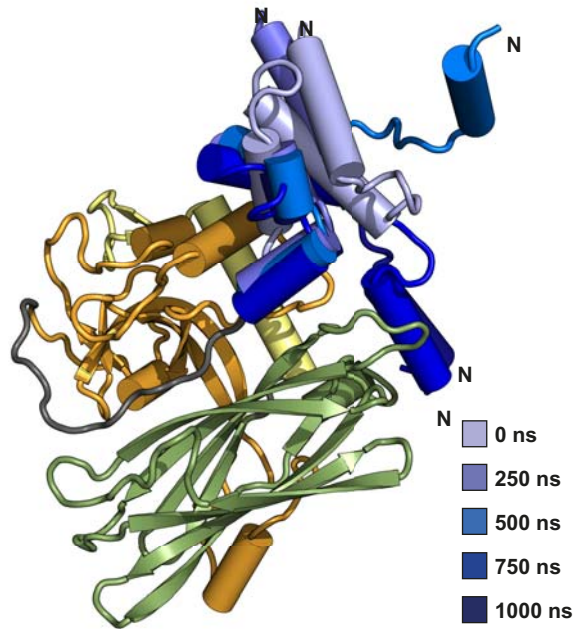

Supplement: Supplementary file 6 — Figure S6: POLDIP2FL molecular dynamics time simulations. (A) root mean square deviation (RMSD, deviation of model over time from reference position) simulation over 100 ns. (B) cartoon representation of NTD dynamics of POLDIP2FL model 41,000 ns simulation. Core structure is model 4 at time 0 ns, with NTD structures superimposed from five time points (N, N‐terminus). [file PRO-30-1196-s001.pdf]

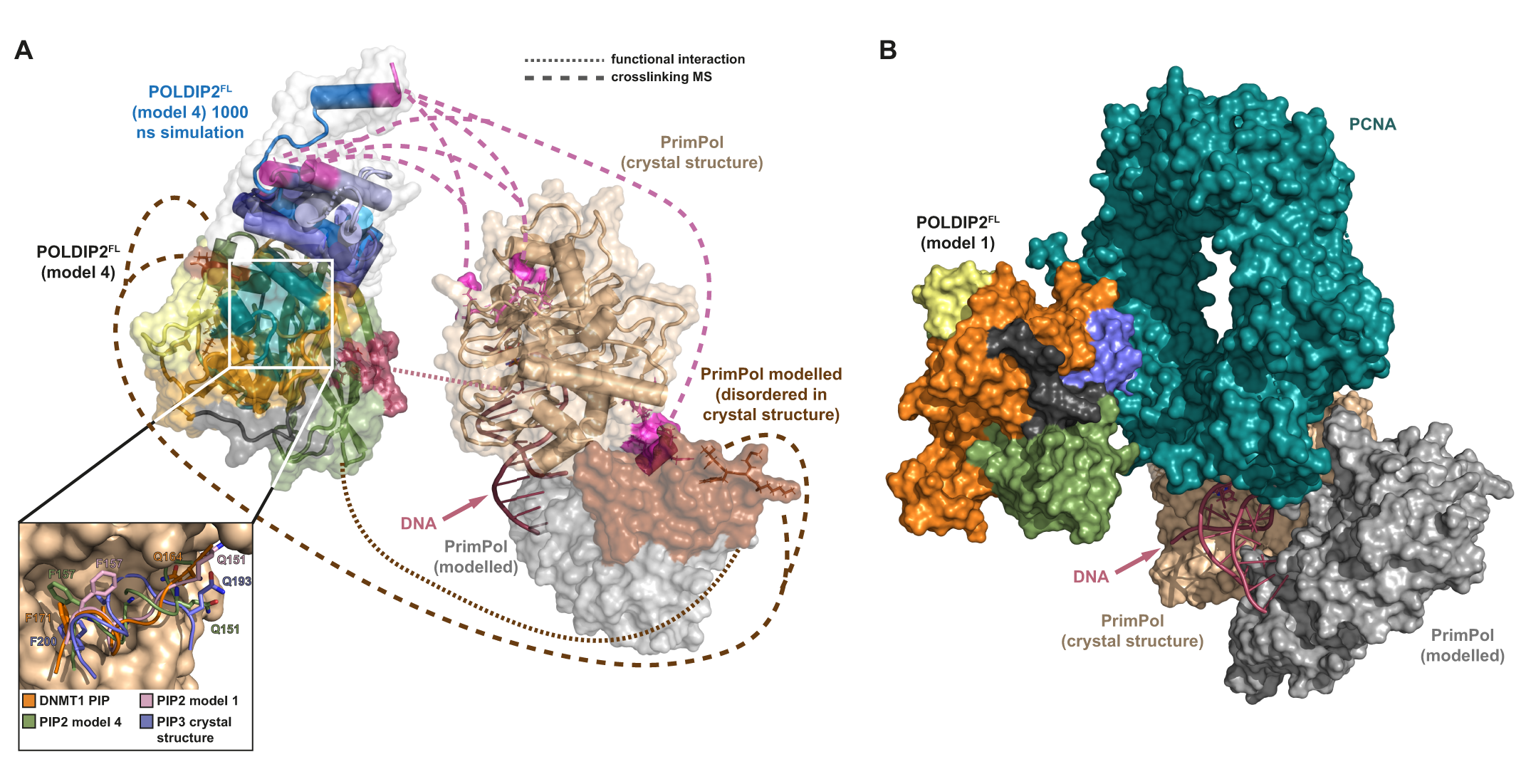

Supplement: Supplementary file 7 — Figure S7: Structural modelling of the POLDIP2FL‐PrimPolFL protein complex. (A) Structural model of POLDIP2FL interactions with PrimPolFL. Molecules are separated to illustrate interactions, with matching colours and connecting lines representing functional (raspberry, dotted lines) or crosslinking mass spectrometric (pink/brown, dashed lines) studies. Protein structures were derived using Robetta from the POLDIP251‐368 crystal structure determined herein (6Z9C), and the PrimPol1‐354 crystal structure (5L2X). POLDIP2FL model 4 (Figure 6) was used as a representative model, with 1,000 ns simulation (Figure S5b) timepoints superimposed for the NTD to illustrate temporal heterogeneity. Dark green/teal, potential PIP boxes in POLDIP2FL model 4. Inset: PIP boxes from the POLDIP251‐368 crystal structure and POLDIP2FL models 1 and 4, superimposed onto the DNMT1 PIP box bound to human PCNA (63KA), with consensus residues represented as sticks. (B) Structural model of human PCNA (63KA) abrogating POLDIP2FL‐PrimPolFL interactions by steric occlusion, with POLDIP2 and PrimPol separated for illustrative purposes. The human PCNA‐DNMT1 PIP‐peptide complex (teal green) was superimposed against the POLDIP2FL model 1 PIP2 box, with POLDIP2FL‐domain colouring as in Figures 1 and 2(a). PrimPol is positioned according to (A) but distanced, to demonstrate mutual exclusivity of PCNA/PrimPol positioning when juxtaposed to POLDIP2. [file PRO-30-1196-s002.tif]
